# Mutual prodrug of cephazolin and benzydamin: 3-[(1-benzyl-1*H*-indazol-3-yl)­oxy]-*N*,*N*-dimethyl­propan-1-aminium 3-{[(5-methyl-1,3,4-thia­diazol-2-yl)sulfan­yl]meth­yl}-8-oxo-7-[(1*H*-tetra­zol-1-yl)acetamido]-5-thia-1-aza­bicyclo­[4.2.0]octane-2-carboxyl­ate (benzydaminium cephazolinate)

**DOI:** 10.1107/S1600536809047941

**Published:** 2009-11-21

**Authors:** Amina Asghar, Mohammad S. Iqbal, M. Nawaz Tahir

**Affiliations:** a Department of Chemistry, Government College University, Lahore, Pakistan; b Department of Physics, University of Sargodha, Sargodha, Pakistan

## Abstract

In the crystal of the title mol­ecular salt, C_19_H_24_N_3_O^+^·C_14_H_13_N_8_O_4_S_3_^−^, the cations and anions are linked by N—H⋯O hydrogen bonds. Short intra­molecular C—H⋯O contacts occur within the anion and inter­molecular C—H⋯O and C—H⋯π bonds help to establish the packing.

## Related literature

Cephazolin, is a first generation cefalosporin antibiotic and benzydamin hydrochloride is a locally acting non-steroidal anti-inflammatory drug with local anaesthetic and analgesic properties. The title compound was prepared as a mutual prodrug for the treatment of infections and inflamatory conditions. For medicinal background to cephazolin, see: Turnbull (1995 ▶). For ring-puckering analysis, see: Cremer & Pople (1975 ▶). For graph-set theory, see: Bernstein *et al.* (1995 ▶).
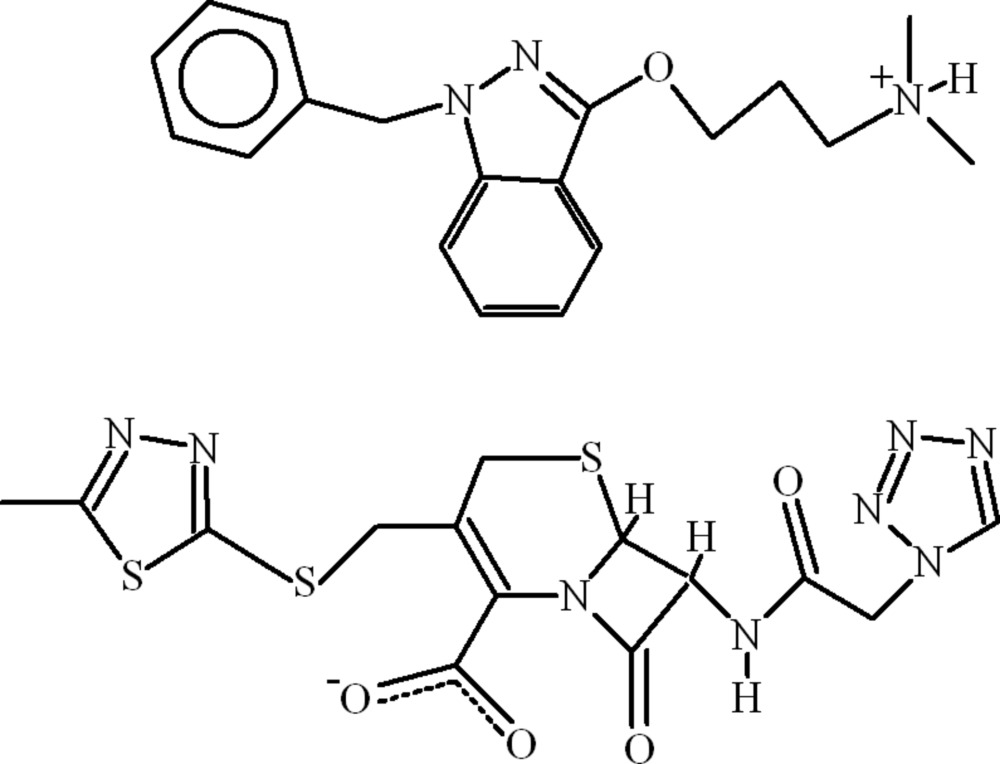


## Experimental

### 

#### Crystal data

C_19_H_24_N_3_O^+^·C_14_H_13_N_8_O_4_S_3_^−^
*M* *_r_* = 763.92
Monoclinic, 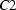

*a* = 44.409 (3) Å
*b* = 7.1777 (5) Å
*c* = 11.2683 (8) Å
β = 90.587 (8)°
*V* = 3591.6 (4) Å^3^
*Z* = 4
Mo *K*α radiation
μ = 0.27 mm^−1^
*T* = 296 K
0.24 × 0.14 × 0.12 mm

#### Data collection

Bruker Kappa APEXII CCD diffractometer
Absorption correction: multi-scan (*SADABS*; Bruker, 2005 ▶) *T*_min_ = 0.957, *T*_max_ = 0.968
34321 measured reflections
6750 independent reflections
4092 reflections with *I* > 2σ(*I*)
*R*_int_ = 0.088

#### Refinement

*R*[*F* ^2^ > 2σ(*F* ^2^)] = 0.050
*wR*(*F* ^2^) = 0.095
*S* = 1.00
6750 reflections
475 parameters
1 restraint
H atoms treated by a mixture of independent and constrained refinement
Δρ_max_ = 0.36 e Å^−3^
Δρ_min_ = −0.23 e Å^−3^
Absolute structure: Flack (1983 ▶), 2918 Friedel Pairs
Flack parameter: −0.07 (7)

### 

Data collection: *APEX2* (Bruker, 2007 ▶); cell refinement: *SAINT* (Bruker, 2007 ▶); data reduction: *SAINT*; program(s) used to solve structure: *SHELXS97* (Sheldrick, 2008 ▶); program(s) used to refine structure: *SHELXL97* (Sheldrick, 2008 ▶); molecular graphics: *ORTEP-3 for Windows* (Farrugia, 1997 ▶) and *PLATON* (Spek, 2009 ▶); software used to prepare material for publication: *WinGX* (Farrugia, 1999 ▶) and *PLATON*.

## Supplementary Material

Crystal structure: contains datablocks global, I. DOI: 10.1107/S1600536809047941/hb5219sup1.cif

Structure factors: contains datablocks I. DOI: 10.1107/S1600536809047941/hb5219Isup2.hkl

Additional supplementary materials:  crystallographic information; 3D view; checkCIF report

## Figures and Tables

**Table 1:** Hydrogen-bond geometry (Å, °)

*D*—H⋯*A*	*D*—H	H⋯*A*	*D*⋯*A*	*D*—H⋯*A*
N3—H3*N*⋯O3^i^	0.96 (4)	1.70 (4)	2.660 (4)	174 (3)
N7—H7*N*⋯O2^ii^	0.86	2.02	2.868 (3)	167
C18—H18*C*⋯O4^iii^	0.96	2.52	3.303 (5)	139
C24—H24*A*⋯N4	0.97	2.41	2.961 (5)	116
C24—H24*B*⋯O3	0.97	2.38	2.882 (4)	112
C32—H32*A*⋯O2^iv^	0.97	2.28	3.218 (4)	164
C32—H32*B*⋯O2^ii^	0.97	2.50	3.318 (4)	142
C19—H19*B*⋯*CgA*	0.96	2.91	3.869 (6)	174
C20—H20*B*⋯*CgD*	0.97	2.72	3.642 (4)	158
C29—H29⋯*CgA* ^ii^	0.98	2.99	3.861 (4)	149

Symmetry codes: (i) 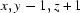; (ii) 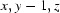; (iii) 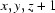; (iv) 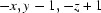. *CgA* and *CgD* are the centroids of the C1–C6 and N8–N11/C33 rings, respectively.

**Fig. 1.**
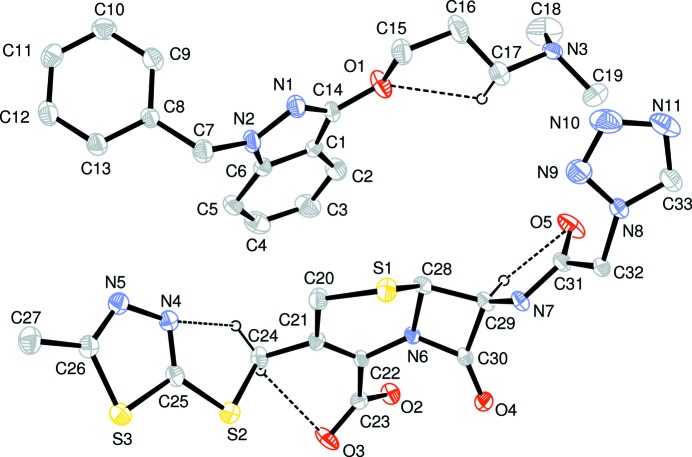
View of (I) with displacement ellipsoids drawn at the 30% probability level.

**Fig. 2.**
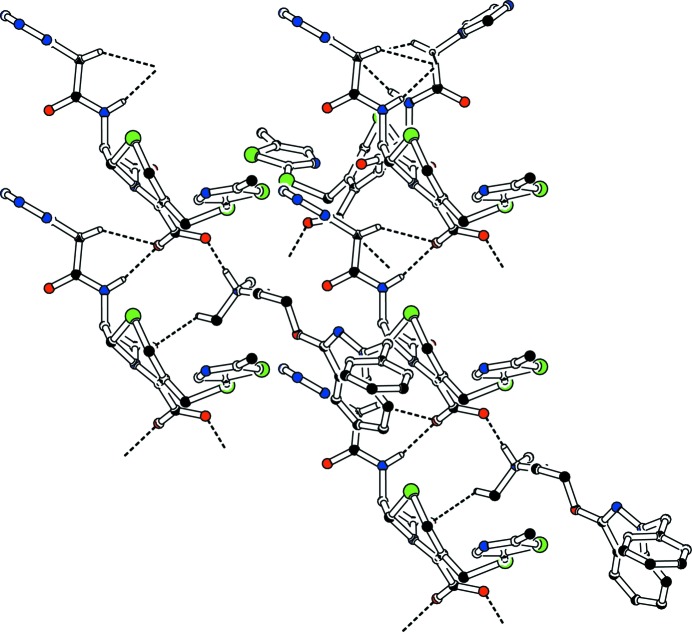
The partial packing of (I), which shows that molecules form polymeric chains and ring motifs.

**Fig. 3.**
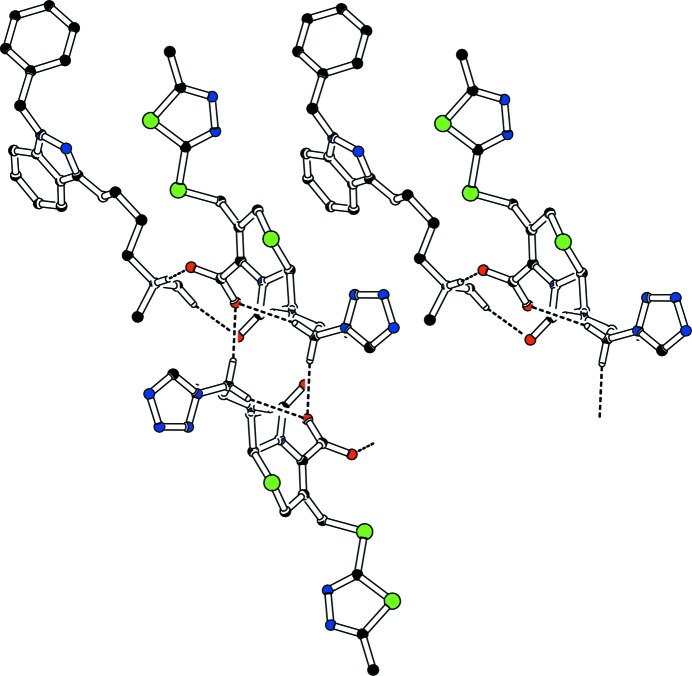
The partial packing of (I), which shows that molecules form polymeric networks with various ring motifs.

## supplementary crystallographic information

### Comment

Cephazolin, is a first generation cefalosporin antibiotic and benzydamin hydrochloride is a locally acting nonsteroidal anti-inflammatory drug with local anaesthetic and analgesic properties providing both rapid and extended pain relief as well as a significant anti-inflammatory treatment for the painful inflammatory conditions of mouth and throat (Turnbull, 1995). The title compound (I, Fig. 1) was prepared as a mutual prodrug for the treatment of infections and inflamatory conditions.

No crystal structure has been found related to cation. In the cation the indazol moiety A (C1–C6/N2/N1/C14) and the benzene ring B (C8–C13) are planar with r.m.s. deviations of 0.0258 and 0.0031 Å respectively, from the respective mean square planes. The dihedral angle between A/B is 71.06 (12)°. In this cation, there exist an intramolecular H-bonding of C—H···O type (Table 1, Fig. 1) forming S(5) ring motif (Bernstein *et al.*, 1995) in the envelop form.

In the anion, two five membered heterocyclic rings C (C25/S3/C26/N5/N4) and D (N8—N9/C33) are planar with r. m. s. deviations of 0.0025 and 0.0014 Å respectively, from the respective mean square planes and the dihedral angle between C/D is 38.58 (14)°. The heterocyclic six membered E (C20/C21/C22/N6/C28/S1) is in the twisted form, with the maximum puckering amplitude Q_T_ = 0.623 (4) Å (Cremer & Pople, 1975) and the four membered ring F (N6/C28–C30) is not planar also. In anion, there exist two S(5) and a S(6) ring motifs (Table 1, Fig. 1). The molecules are stabilized in the form of polymeric chains forming *R*_2_^1^(6), *R*_4_^2^(8), *R*_2_^2^(10) and other ring motifs (Fig. 2 & Fig. 3).

### Experimental

Cefazolin sodium (0.238 g; 0.1 mol) and benzydamine hydrochloride (0.173 g; 0.01 mol) were dissolved separately in distilled water (50 ml). Ten milliliter of each solution were mixed and left for two days. The colourless needles of (I) thus obtained were filtered out by suction, washed with distilled water and dried under vacuum.

### Refinement

The coordinates of H3N were refined. The H-atoms were positioned geometrically (O–H = 0.82 Å, N–H = 0.86 Å, C–H = 0.93–0.97 Å) and refined as riding with *U*_iso_(H) = 1.2*U*_eq_(carrier) or 1.5*U*_eq_(methyl C).

### Crystal data

**Table:**

C_19_H_24_N_3_O^+^·C_14_H_13_N_8_O_4_S_3_^−^	*F*(000) = 1600
*M**_r_* = 763.92	*D*_x_ = 1.413 Mg m^−^^3^
Monoclinic, *C*2	Mo *K*α radiation, λ = 0.71073 Å
Hall symbol: C 2y	Cell parameters from 3824 reflections
*a* = 44.409 (3) Å	θ = 2.6–26.0°
*b* = 7.1777 (5) Å	µ = 0.27 mm^−^^1^
*c* = 11.2683 (8) Å	*T* = 296 K
β = 90.587 (8)°	Cut needle, colourless
*V* = 3591.6 (4) Å^3^	0.24 × 0.14 × 0.12 mm
*Z* = 4	

### Data collection

**Table:**

Bruker Kappa APEXII CCD diffractometer	6750 independent reflections
Radiation source: fine-focus sealed tube	4092 reflections with *I* > 2σ(*I*)
graphite	*R*_int_ = 0.088
Detector resolution: 7.60 pixels mm^-1^	θ_max_ = 26.0°, θ_min_ = 2.6°
ω scans	*h* = −54→54
Absorption correction: multi-scan (*SADABS*; Bruker, 2005)	*k* = −8→8
*T*_min_ = 0.957, *T*_max_ = 0.968	*l* = −13→13
34321 measured reflections	

### Refinement

**Table:**

Refinement on *F*^2^	Secondary atom site location: difference Fourier map
Least-squares matrix: full	Hydrogen site location: inferred from neighbouring sites
*R*[*F*^2^ > 2σ(*F*^2^)] = 0.050	H atoms treated by a mixture of independent and constrained refinement
*wR*(*F*^2^) = 0.095	*w* = 1/[σ^2^(*F*_o_^2^) + (0.0342*P*)^2^] where *P* = (*F*_o_^2^ + 2*F*_c_^2^)/3
*S* = 1.00	(Δ/σ)_max_ < 0.001
6750 reflections	Δρ_max_ = 0.36 e Å^−^^3^
475 parameters	Δρ_min_ = −0.23 e Å^−^^3^
1 restraint	Absolute structure: Flack (1983), 2918 Friedal Pairs
Primary atom site location: structure-invariant direct methods	Flack parameter: −0.07 (7)

### Special details

**Table:**

Geometry. Bond distances, angles *etc*. have been calculated using the rounded fractional coordinates. All su's are estimated from the variances of the (full) variance-covariance matrix. The cell e.s.d.'s are taken into account in the estimation of distances, angles and torsion angles
Refinement. Refinement of *F*^2^ against ALL reflections. The weighted *R*-factor *wR* and goodness of fit *S* are based on *F*^2^, conventional *R*-factors *R* are based on *F*, with *F* set to zero for negative *F*^2^. The threshold expression of *F*^2^ > σ(*F*^2^) is used only for calculating *R*-factors(gt) *etc*. and is not relevant to the choice of reflections for refinement. *R*-factors based on *F*^2^ are statistically about twice as large as those based on *F*, and *R*- factors based on ALL data will be even larger.

### Fractional atomic coordinates and isotropic or equivalent isotropic displacement parameters (Å^2^)

**Table:**

	*x*	*y*	*z*	*U*_iso_*/*U*_eq_	
O1	0.14103 (6)	0.2863 (4)	0.9240 (3)	0.0552 (11)	
N1	0.18241 (7)	0.2542 (4)	0.7971 (3)	0.0420 (12)	
N2	0.19416 (7)	0.3698 (5)	0.7111 (3)	0.0433 (12)	
N3	0.06627 (7)	0.0749 (5)	1.1012 (3)	0.0372 (11)	
C1	0.15436 (8)	0.5181 (5)	0.7788 (3)	0.0340 (12)	
C2	0.13236 (9)	0.6570 (6)	0.7813 (4)	0.0510 (17)	
C3	0.13442 (11)	0.8000 (7)	0.7038 (4)	0.0640 (17)	
C4	0.15844 (11)	0.8115 (6)	0.6251 (4)	0.0610 (19)	
C5	0.18016 (10)	0.6769 (6)	0.6189 (3)	0.0490 (17)	
C6	0.17762 (8)	0.5263 (5)	0.6969 (3)	0.0370 (12)	
C7	0.21920 (9)	0.3080 (6)	0.6398 (3)	0.0463 (17)	
C8	0.24923 (8)	0.3866 (5)	0.6760 (3)	0.0353 (12)	
C9	0.25878 (9)	0.3855 (6)	0.7933 (4)	0.0466 (17)	
C10	0.28704 (10)	0.4478 (6)	0.8244 (4)	0.0520 (17)	
C11	0.30587 (9)	0.5147 (6)	0.7391 (4)	0.0484 (17)	
C12	0.29672 (9)	0.5182 (6)	0.6231 (4)	0.0492 (17)	
C13	0.26851 (9)	0.4546 (6)	0.5911 (3)	0.0450 (17)	
C14	0.15916 (9)	0.3469 (5)	0.8366 (3)	0.0393 (16)	
C15	0.14483 (9)	0.1004 (6)	0.9660 (4)	0.0546 (17)	
C16	0.12086 (10)	0.0662 (8)	1.0548 (4)	0.0699 (19)	
C17	0.09024 (9)	0.0869 (7)	1.0079 (4)	0.0636 (19)	
C18	0.06411 (12)	0.2420 (7)	1.1765 (4)	0.089 (3)	
C19	0.03700 (9)	0.0436 (8)	1.0420 (4)	0.0718 (19)	
S1	0.10575 (2)	0.19304 (14)	0.49823 (9)	0.0428 (3)	
S2	0.14745 (2)	0.56694 (16)	0.18123 (9)	0.0463 (4)	
S3	0.20738 (2)	0.44836 (15)	0.08659 (9)	0.0456 (4)	
O2	0.05024 (6)	0.8249 (3)	0.3777 (2)	0.0384 (9)	
O3	0.08047 (6)	0.7715 (4)	0.2253 (2)	0.0547 (11)	
O4	0.02066 (6)	0.4136 (4)	0.3861 (2)	0.0405 (9)	
O5	0.02552 (7)	0.0728 (4)	0.7592 (2)	0.0587 (13)	
N4	0.19862 (7)	0.4956 (4)	0.3076 (3)	0.0406 (12)	
N5	0.22865 (7)	0.4435 (4)	0.2966 (3)	0.0427 (12)	
N6	0.06812 (6)	0.4815 (4)	0.4717 (2)	0.0279 (10)	
N7	0.03965 (6)	0.0819 (4)	0.5676 (2)	0.0315 (10)	
N8	0.02949 (7)	−0.3125 (4)	0.7545 (3)	0.0325 (11)	
N9	0.05801 (8)	−0.3400 (5)	0.7855 (3)	0.0603 (16)	
N10	0.05818 (10)	−0.4303 (7)	0.8833 (4)	0.0751 (17)	
N11	0.02978 (11)	−0.4641 (5)	0.9184 (3)	0.0663 (16)	
C20	0.13044 (8)	0.3791 (5)	0.4529 (3)	0.0440 (14)	
C21	0.11584 (8)	0.5428 (5)	0.3900 (3)	0.0317 (12)	
C22	0.08645 (8)	0.5831 (5)	0.3948 (3)	0.0293 (12)	
C23	0.07099 (9)	0.7410 (5)	0.3273 (3)	0.0330 (12)	
C24	0.13782 (8)	0.6622 (5)	0.3251 (3)	0.0396 (14)	
C25	0.18490 (8)	0.5045 (5)	0.2061 (3)	0.0376 (12)	
C26	0.23635 (9)	0.4134 (5)	0.1884 (3)	0.0407 (14)	
C27	0.26722 (9)	0.3571 (6)	0.1520 (4)	0.0570 (17)	
C28	0.07883 (8)	0.3496 (5)	0.5621 (3)	0.0347 (12)	
C29	0.04609 (8)	0.2771 (5)	0.5704 (3)	0.0323 (12)	
C30	0.04024 (8)	0.3968 (5)	0.4591 (3)	0.0289 (12)	
C31	0.02943 (8)	−0.0044 (5)	0.6644 (3)	0.0340 (12)	
C32	0.02179 (8)	−0.2078 (5)	0.6480 (3)	0.0337 (12)	
C33	0.01254 (10)	−0.3891 (5)	0.8368 (4)	0.0479 (17)	
H2	0.11664	0.65124	0.83513	0.0612*	
H3	0.11965	0.89190	0.70279	0.0762*	
H3N	0.0701 (8)	−0.035 (5)	1.148 (3)	0.0446*	
H4	0.15971	0.91434	0.57520	0.0732*	
H5	0.19589	0.68514	0.56533	0.0588*	
H7A	0.21521	0.34164	0.55778	0.0555*	
H7B	0.22030	0.17314	0.64381	0.0555*	
H9	0.24592	0.34200	0.85174	0.0560*	
H10	0.29334	0.44458	0.90336	0.0622*	
H11	0.32494	0.55774	0.76019	0.0578*	
H12	0.30960	0.56374	0.56540	0.0593*	
H13	0.26244	0.45759	0.51190	0.0541*	
H15A	0.16458	0.08557	1.00236	0.0653*	
H15B	0.14299	0.01281	0.90075	0.0653*	
H16A	0.12321	−0.05902	1.08589	0.0837*	
H16B	0.12364	0.15230	1.12035	0.0837*	
H17A	0.08661	−0.00932	0.94905	0.0766*	
H17B	0.08860	0.20648	0.96821	0.0766*	
H18A	0.05950	0.34834	1.12796	0.1331*	
H18B	0.08295	0.26181	1.21710	0.1331*	
H18C	0.04846	0.22478	1.23366	0.1331*	
H19A	0.03248	0.14682	0.99069	0.1076*	
H19B	0.02159	0.03237	1.10070	0.1076*	
H19C	0.03785	−0.06890	0.99605	0.1076*	
H7N	0.04237	0.01975	0.50338	0.0378*	
H20A	0.14087	0.42565	0.52288	0.0529*	
H20B	0.14552	0.32694	0.40083	0.0529*	
H24A	0.15602	0.67492	0.37270	0.0474*	
H24B	0.12927	0.78543	0.31426	0.0474*	
H27A	0.27708	0.29376	0.21663	0.0852*	
H27B	0.27858	0.46598	0.13134	0.0852*	
H27C	0.26590	0.27544	0.08474	0.0852*	
H28	0.08607	0.41003	0.63503	0.0418*	
H29	0.03622	0.33403	0.63884	0.0385*	
H32A	0.00044	−0.22075	0.63099	0.0402*	
H32B	0.03281	−0.25736	0.58121	0.0402*	
H33	−0.00840	−0.38952	0.83662	0.0575*	

### Atomic displacement parameters (Å^2^)

**Table:**

	*U*^11^	*U*^22^	*U*^33^	*U*^12^	*U*^13^	*U*^23^
O1	0.058 (2)	0.0430 (17)	0.065 (2)	0.0076 (15)	0.0274 (16)	0.0174 (15)
N1	0.036 (2)	0.044 (2)	0.046 (2)	0.0012 (17)	0.0080 (18)	0.0076 (17)
N2	0.031 (2)	0.052 (2)	0.047 (2)	0.0036 (18)	0.0123 (17)	0.0080 (18)
N3	0.039 (2)	0.043 (2)	0.0295 (19)	0.0030 (18)	0.0015 (16)	0.0090 (17)
C1	0.030 (2)	0.037 (2)	0.035 (2)	−0.0035 (19)	−0.0037 (19)	0.0030 (19)
C2	0.049 (3)	0.050 (3)	0.054 (3)	0.008 (2)	0.007 (2)	0.009 (2)
C3	0.070 (3)	0.064 (3)	0.058 (3)	0.021 (3)	0.005 (3)	0.014 (3)
C4	0.082 (4)	0.048 (3)	0.053 (3)	0.003 (3)	0.002 (3)	0.013 (2)
C5	0.054 (3)	0.054 (3)	0.039 (3)	−0.015 (3)	0.001 (2)	0.010 (2)
C6	0.034 (2)	0.040 (2)	0.037 (2)	−0.006 (2)	−0.004 (2)	0.002 (2)
C7	0.042 (3)	0.051 (3)	0.046 (3)	−0.002 (2)	0.006 (2)	−0.003 (2)
C8	0.034 (2)	0.036 (2)	0.036 (2)	0.0023 (19)	0.005 (2)	0.003 (2)
C9	0.049 (3)	0.049 (3)	0.042 (3)	−0.007 (2)	0.008 (2)	0.001 (2)
C10	0.059 (3)	0.056 (3)	0.041 (3)	−0.008 (3)	−0.005 (2)	−0.002 (2)
C11	0.040 (3)	0.042 (3)	0.063 (3)	0.007 (2)	−0.003 (2)	0.000 (2)
C12	0.041 (3)	0.052 (3)	0.055 (3)	0.004 (2)	0.014 (2)	0.009 (2)
C13	0.042 (3)	0.051 (3)	0.042 (3)	0.004 (2)	0.006 (2)	0.004 (2)
C14	0.031 (2)	0.047 (3)	0.040 (3)	−0.006 (2)	0.006 (2)	0.009 (2)
C15	0.042 (3)	0.054 (3)	0.068 (3)	0.005 (2)	0.010 (2)	0.015 (3)
C16	0.054 (3)	0.071 (3)	0.085 (4)	0.004 (3)	0.023 (3)	0.042 (3)
C17	0.051 (3)	0.082 (4)	0.058 (3)	−0.010 (3)	0.006 (3)	0.024 (3)
C18	0.128 (5)	0.061 (4)	0.077 (4)	0.019 (3)	−0.010 (4)	−0.030 (3)
C19	0.052 (3)	0.101 (4)	0.062 (3)	−0.009 (3)	−0.015 (2)	0.006 (3)
S1	0.0374 (6)	0.0314 (5)	0.0596 (7)	0.0060 (5)	0.0004 (5)	0.0109 (5)
S2	0.0375 (6)	0.0565 (7)	0.0448 (7)	−0.0009 (6)	0.0007 (5)	0.0019 (6)
S3	0.0463 (7)	0.0527 (7)	0.0380 (6)	0.0001 (6)	0.0054 (5)	−0.0031 (5)
O2	0.0384 (16)	0.0316 (15)	0.0452 (17)	0.0089 (13)	0.0084 (14)	0.0012 (13)
O3	0.067 (2)	0.0582 (19)	0.0393 (17)	0.0184 (16)	0.0200 (16)	0.0246 (15)
O4	0.0324 (15)	0.0485 (17)	0.0406 (16)	−0.0028 (13)	−0.0041 (14)	0.0086 (14)
O5	0.106 (3)	0.0393 (17)	0.0313 (16)	−0.0094 (17)	0.0191 (16)	−0.0010 (15)
N4	0.037 (2)	0.046 (2)	0.039 (2)	0.0003 (16)	0.0038 (16)	0.0039 (16)
N5	0.038 (2)	0.047 (2)	0.043 (2)	0.0016 (18)	0.0012 (17)	0.0032 (18)
N6	0.0282 (18)	0.0253 (17)	0.0302 (17)	−0.0001 (14)	0.0004 (14)	0.0099 (14)
N7	0.0414 (19)	0.0239 (17)	0.0293 (18)	−0.0033 (15)	0.0101 (15)	0.0011 (15)
N8	0.038 (2)	0.0287 (16)	0.0308 (19)	−0.0024 (16)	0.0055 (16)	0.0022 (16)
N9	0.044 (2)	0.080 (3)	0.057 (3)	0.010 (2)	0.000 (2)	0.016 (2)
N10	0.074 (3)	0.090 (3)	0.061 (3)	0.028 (3)	−0.009 (2)	0.022 (3)
N11	0.096 (3)	0.059 (3)	0.044 (2)	0.001 (3)	0.003 (2)	0.020 (2)
C20	0.036 (2)	0.043 (2)	0.053 (3)	−0.003 (2)	0.000 (2)	0.011 (2)
C21	0.031 (2)	0.027 (2)	0.037 (2)	−0.0021 (18)	0.0007 (18)	0.0008 (18)
C22	0.030 (2)	0.028 (2)	0.030 (2)	−0.0067 (18)	0.0040 (17)	0.0024 (18)
C23	0.036 (2)	0.028 (2)	0.035 (2)	−0.0032 (18)	−0.005 (2)	0.0028 (19)
C24	0.035 (2)	0.034 (2)	0.050 (3)	−0.0034 (19)	0.0070 (19)	0.004 (2)
C25	0.040 (2)	0.035 (2)	0.038 (2)	−0.0023 (19)	0.007 (2)	0.0035 (19)
C26	0.037 (2)	0.041 (2)	0.044 (3)	−0.001 (2)	0.003 (2)	−0.003 (2)
C27	0.044 (3)	0.062 (3)	0.065 (3)	0.006 (2)	0.008 (2)	0.001 (2)
C28	0.039 (2)	0.035 (2)	0.030 (2)	0.0001 (19)	0.0014 (19)	0.0055 (18)
C29	0.039 (2)	0.029 (2)	0.029 (2)	0.0000 (19)	0.0081 (18)	0.0008 (18)
C30	0.029 (2)	0.029 (2)	0.029 (2)	0.0037 (19)	0.0095 (19)	−0.0001 (18)
C31	0.036 (2)	0.036 (2)	0.030 (2)	−0.0018 (19)	0.0035 (19)	0.0063 (19)
C32	0.036 (2)	0.033 (2)	0.032 (2)	−0.0091 (18)	0.0000 (18)	0.0061 (18)
C33	0.055 (3)	0.048 (3)	0.041 (3)	−0.007 (2)	0.009 (2)	0.008 (2)

### Geometric parameters (Å, °)

**Table:**

S1—C20	1.805 (4)	C11—C12	1.365 (6)
S1—C28	1.797 (4)	C12—C13	1.378 (6)
S2—C24	1.815 (4)	C15—C16	1.489 (6)
S2—C25	1.742 (4)	C16—C17	1.461 (6)
S3—C26	1.733 (4)	C2—H2	0.9300
S3—C25	1.732 (4)	C3—H3	0.9300
O1—C15	1.425 (5)	C4—H4	0.9300
O1—C14	1.350 (5)	C5—H5	0.9300
O2—C23	1.243 (5)	C7—H7B	0.9700
O3—C23	1.247 (4)	C7—H7A	0.9700
O4—C30	1.197 (4)	C9—H9	0.9300
O5—C31	1.217 (4)	C10—H10	0.9300
N1—N2	1.382 (5)	C11—H11	0.9300
N1—C14	1.310 (5)	C12—H12	0.9300
N2—C7	1.448 (5)	C13—H13	0.9300
N2—C6	1.351 (5)	C15—H15B	0.9700
N3—C17	1.506 (5)	C15—H15A	0.9700
N3—C18	1.473 (6)	C16—H16A	0.9700
N3—C19	1.472 (5)	C16—H16B	0.9700
N3—H3N	0.96 (4)	C17—H17A	0.9700
N4—N5	1.392 (4)	C17—H17B	0.9700
N4—C25	1.292 (5)	C18—H18B	0.9600
N5—C26	1.288 (5)	C18—H18A	0.9600
N6—C28	1.466 (4)	C18—H18C	0.9600
N6—C30	1.385 (4)	C19—H19C	0.9600
N6—C22	1.400 (4)	C19—H19B	0.9600
N7—C31	1.338 (4)	C19—H19A	0.9600
N7—C29	1.430 (5)	C20—C21	1.515 (5)
N8—N9	1.325 (5)	C21—C24	1.496 (5)
N8—C33	1.320 (5)	C21—C22	1.339 (5)
N8—C32	1.454 (5)	C22—C23	1.524 (5)
N9—N10	1.279 (6)	C26—C27	1.491 (6)
N10—N11	1.348 (7)	C28—C29	1.548 (5)
N11—C33	1.307 (6)	C29—C30	1.540 (5)
N7—H7N	0.8600	C31—C32	1.510 (5)
C1—C14	1.406 (5)	C20—H20A	0.9700
C1—C2	1.396 (5)	C20—H20B	0.9700
C1—C6	1.393 (5)	C24—H24A	0.9700
C2—C3	1.351 (7)	C24—H24B	0.9700
C3—C4	1.397 (7)	C27—H27A	0.9600
C4—C5	1.368 (6)	C27—H27B	0.9600
C5—C6	1.399 (5)	C27—H27C	0.9600
C7—C8	1.501 (5)	C28—H28	0.9800
C8—C9	1.384 (6)	C29—H29	0.9800
C8—C13	1.380 (5)	C32—H32A	0.9700
C9—C10	1.374 (6)	C32—H32B	0.9700
C10—C11	1.368 (6)	C33—H33	0.9300
			
C20—S1—C28	93.40 (17)	C15—C16—H16B	109.00
C24—S2—C25	100.75 (17)	C17—C16—H16B	109.00
C25—S3—C26	87.07 (17)	H16A—C16—H16B	108.00
C14—O1—C15	118.3 (3)	C17—C16—H16A	109.00
N2—N1—C14	103.8 (3)	C16—C17—H17A	109.00
C6—N2—C7	127.5 (3)	C16—C17—H17B	109.00
N1—N2—C6	111.9 (3)	H17A—C17—H17B	108.00
N1—N2—C7	120.2 (3)	N3—C17—H17B	109.00
C17—N3—C18	114.0 (4)	N3—C17—H17A	109.00
C17—N3—C19	108.6 (3)	N3—C18—H18B	109.00
C18—N3—C19	108.8 (4)	N3—C18—H18A	110.00
C17—N3—H3N	108 (2)	H18A—C18—H18C	109.00
C18—N3—H3N	111 (2)	N3—C18—H18C	109.00
C19—N3—H3N	106 (2)	H18A—C18—H18B	109.00
N5—N4—C25	112.2 (3)	H18B—C18—H18C	109.00
N4—N5—C26	113.2 (3)	N3—C19—H19B	109.00
C22—N6—C30	133.6 (3)	N3—C19—H19C	109.00
C22—N6—C28	125.4 (3)	H19A—C19—H19C	109.00
C28—N6—C30	94.1 (3)	H19B—C19—H19C	109.00
C29—N7—C31	120.3 (3)	H19A—C19—H19B	109.00
N9—N8—C32	120.7 (3)	N3—C19—H19A	109.00
C32—N8—C33	131.6 (3)	S1—C20—C21	116.6 (3)
N9—N8—C33	107.6 (3)	C22—C21—C24	122.5 (3)
N8—N9—N10	107.5 (3)	C20—C21—C22	124.2 (3)
N9—N10—N11	110.3 (4)	C20—C21—C24	113.3 (3)
N10—N11—C33	105.2 (4)	N6—C22—C23	115.8 (3)
C29—N7—H7N	120.00	N6—C22—C21	119.1 (3)
C31—N7—H7N	120.00	C21—C22—C23	125.1 (3)
C6—C1—C14	103.5 (3)	O2—C23—C22	117.7 (3)
C2—C1—C6	120.5 (3)	O2—C23—O3	126.5 (3)
C2—C1—C14	135.8 (4)	O3—C23—C22	115.8 (3)
C1—C2—C3	118.5 (4)	S2—C24—C21	112.5 (2)
C2—C3—C4	120.8 (4)	S2—C25—N4	126.6 (3)
C3—C4—C5	122.3 (4)	S2—C25—S3	119.5 (2)
C4—C5—C6	116.9 (4)	S3—C25—N4	114.0 (3)
N2—C6—C1	107.0 (3)	S3—C26—C27	122.4 (3)
C1—C6—C5	120.9 (3)	S3—C26—N5	113.6 (3)
N2—C6—C5	132.1 (3)	N5—C26—C27	124.0 (4)
N2—C7—C8	114.8 (3)	N6—C28—C29	87.8 (2)
C7—C8—C9	121.4 (3)	S1—C28—C29	116.3 (2)
C7—C8—C13	120.0 (3)	S1—C28—N6	109.8 (2)
C9—C8—C13	118.6 (3)	N7—C29—C28	121.0 (3)
C8—C9—C10	120.8 (4)	N7—C29—C30	119.8 (3)
C9—C10—C11	119.9 (4)	C28—C29—C30	85.1 (3)
C10—C11—C12	120.1 (4)	O4—C30—N6	132.0 (3)
C11—C12—C13	120.4 (4)	N6—C30—C29	91.0 (3)
C8—C13—C12	120.3 (3)	O4—C30—C29	137.0 (3)
N1—C14—C1	113.8 (3)	O5—C31—C32	120.9 (3)
O1—C14—N1	124.2 (3)	O5—C31—N7	123.9 (3)
O1—C14—C1	122.1 (3)	N7—C31—C32	115.2 (3)
O1—C15—C16	107.1 (4)	N8—C32—C31	110.4 (3)
C15—C16—C17	114.2 (4)	N8—C33—N11	109.4 (4)
N3—C17—C16	113.8 (4)	S1—C20—H20A	108.00
C1—C2—H2	121.00	S1—C20—H20B	108.00
C3—C2—H2	121.00	C21—C20—H20A	108.00
C4—C3—H3	120.00	C21—C20—H20B	108.00
C2—C3—H3	120.00	H20A—C20—H20B	107.00
C3—C4—H4	119.00	S2—C24—H24A	109.00
C5—C4—H4	119.00	S2—C24—H24B	109.00
C6—C5—H5	122.00	C21—C24—H24A	109.00
C4—C5—H5	122.00	C21—C24—H24B	109.00
N2—C7—H7A	109.00	H24A—C24—H24B	108.00
C8—C7—H7B	109.00	C26—C27—H27A	109.00
N2—C7—H7B	109.00	C26—C27—H27B	109.00
C8—C7—H7A	109.00	C26—C27—H27C	110.00
H7A—C7—H7B	108.00	H27A—C27—H27B	109.00
C10—C9—H9	120.00	H27A—C27—H27C	109.00
C8—C9—H9	120.00	H27B—C27—H27C	109.00
C9—C10—H10	120.00	S1—C28—H28	113.00
C11—C10—H10	120.00	N6—C28—H28	113.00
C12—C11—H11	120.00	C29—C28—H28	113.00
C10—C11—H11	120.00	N7—C29—H29	110.00
C13—C12—H12	120.00	C28—C29—H29	110.00
C11—C12—H12	120.00	C30—C29—H29	110.00
C8—C13—H13	120.00	N8—C32—H32A	110.00
C12—C13—H13	120.00	N8—C32—H32B	110.00
O1—C15—H15B	110.00	C31—C32—H32A	110.00
C16—C15—H15A	110.00	C31—C32—H32B	110.00
O1—C15—H15A	110.00	H32A—C32—H32B	108.00
H15A—C15—H15B	109.00	N8—C33—H33	125.00
C16—C15—H15B	110.00	N11—C33—H33	125.00
C15—C16—H16A	109.00		
			
C20—S1—C28—C29	155.3 (3)	N8—N9—N10—N11	0.1 (5)
C20—S1—C28—N6	57.8 (3)	N9—N10—N11—C33	−0.1 (5)
C28—S1—C20—C21	−47.1 (3)	N10—N11—C33—N8	0.2 (5)
C24—S2—C25—N4	12.6 (4)	C2—C1—C6—N2	−176.6 (3)
C24—S2—C25—S3	−167.0 (2)	C2—C1—C6—C5	2.6 (6)
C25—S2—C24—C21	−111.3 (3)	C14—C1—C2—C3	−175.0 (4)
C25—S3—C26—C27	−179.3 (3)	C14—C1—C6—C5	178.4 (3)
C26—S3—C25—N4	−0.1 (3)	C2—C1—C14—O1	−5.2 (7)
C25—S3—C26—N5	−0.4 (3)	C14—C1—C6—N2	−0.8 (4)
C26—S3—C25—S2	179.6 (2)	C6—C1—C2—C3	−0.9 (6)
C14—O1—C15—C16	−176.1 (3)	C6—C1—C14—N1	−0.5 (4)
C15—O1—C14—C1	171.1 (3)	C2—C1—C14—N1	174.4 (4)
C15—O1—C14—N1	−8.4 (5)	C6—C1—C14—O1	179.9 (3)
N2—N1—C14—C1	1.4 (4)	C1—C2—C3—C4	−1.7 (7)
C14—N1—N2—C7	−175.3 (3)	C2—C3—C4—C5	2.7 (7)
N2—N1—C14—O1	−179.0 (3)	C3—C4—C5—C6	−1.0 (6)
C14—N1—N2—C6	−1.9 (4)	C4—C5—C6—C1	−1.6 (6)
N1—N2—C6—C5	−177.3 (4)	C4—C5—C6—N2	177.3 (4)
C7—N2—C6—C5	−4.6 (7)	N2—C7—C8—C9	48.2 (5)
N1—N2—C7—C8	−102.2 (4)	N2—C7—C8—C13	−134.6 (4)
C6—N2—C7—C8	85.6 (5)	C7—C8—C13—C12	−176.7 (4)
C7—N2—C6—C1	174.5 (3)	C9—C8—C13—C12	0.6 (6)
N1—N2—C6—C1	1.7 (4)	C13—C8—C9—C10	−1.1 (6)
C19—N3—C17—C16	−164.0 (4)	C7—C8—C9—C10	176.1 (4)
C18—N3—C17—C16	74.5 (5)	C8—C9—C10—C11	1.1 (6)
C25—N4—N5—C26	−0.7 (4)	C9—C10—C11—C12	−0.5 (7)
N5—N4—C25—S3	0.4 (4)	C10—C11—C12—C13	0.0 (7)
N5—N4—C25—S2	−179.2 (3)	C11—C12—C13—C8	0.0 (6)
N4—N5—C26—S3	0.6 (4)	O1—C15—C16—C17	59.5 (5)
N4—N5—C26—C27	179.6 (3)	C15—C16—C17—N3	−173.0 (4)
C30—N6—C22—C21	−133.6 (4)	S1—C20—C21—C22	19.2 (5)
C30—N6—C22—C23	49.7 (5)	S1—C20—C21—C24	−163.3 (2)
C28—N6—C22—C23	−167.3 (3)	C20—C21—C22—N6	6.9 (5)
C30—N6—C28—C29	−11.3 (3)	C20—C21—C22—C23	−176.7 (3)
C22—N6—C28—S1	−48.2 (4)	C20—C21—C24—S2	81.3 (3)
C22—N6—C28—C29	−165.4 (3)	C22—C21—C24—S2	−101.2 (4)
C22—N6—C30—O4	−16.5 (7)	C24—C21—C22—N6	−170.3 (3)
C28—N6—C22—C21	9.4 (5)	C24—C21—C22—C23	6.0 (6)
C30—N6—C28—S1	106.0 (2)	N6—C22—C23—O2	32.8 (5)
C22—N6—C30—C29	161.9 (4)	C21—C22—C23—O3	37.0 (5)
C28—N6—C30—O4	−167.1 (4)	N6—C22—C23—O3	−146.5 (3)
C28—N6—C30—C29	11.3 (3)	C21—C22—C23—O2	−143.7 (4)
C29—N7—C31—O5	−1.4 (5)	S1—C28—C29—N7	21.1 (4)
C31—N7—C29—C30	−145.3 (3)	S1—C28—C29—C30	−100.9 (3)
C29—N7—C31—C32	176.6 (3)	N6—C28—C29—N7	132.1 (3)
C31—N7—C29—C28	111.5 (4)	N6—C28—C29—C30	10.1 (2)
N9—N8—C32—C31	−69.8 (4)	N7—C29—C30—O4	44.4 (6)
N9—N8—C33—N11	−0.1 (4)	N7—C29—C30—N6	−133.9 (3)
C32—N8—C33—N11	−177.9 (3)	C28—C29—C30—O4	167.5 (5)
C33—N8—N9—N10	0.0 (4)	C28—C29—C30—N6	−10.7 (3)
C32—N8—N9—N10	178.1 (4)	O5—C31—C32—N8	−38.1 (5)
C33—N8—C32—C31	107.8 (4)	N7—C31—C32—N8	143.9 (3)

### Hydrogen-bond geometry (Å, °)

**Table:**

*D*—H···*A*	*D*—H	H···*A*	*D*···*A*	*D*—H···*A*
N3—H3N···O3^i^	0.96 (4)	1.70 (4)	2.660 (4)	174 (3)
N7—H7N···O2^ii^	0.8600	2.0200	2.868 (3)	167.00
C18—H18C···O4^iii^	0.9600	2.5200	3.303 (5)	139.00
C24—H24A···N4	0.9700	2.4100	2.961 (5)	116.00
C24—H24B···O3	0.9700	2.3800	2.882 (4)	112.00
C32—H32A···O2^iv^	0.9700	2.2800	3.218 (4)	164.00
C32—H32B···O2^ii^	0.9700	2.5000	3.318 (4)	142.00
C19—H19B···CgA	0.96	2.91	3.869 (6)	174
C20—H20B···CgD	0.97	2.72	3.642 (4)	158
C29—H29···CgA^ii^	0.98	2.99	3.861 (4)	149

Symmetry codes: (i) *x*, *y*−1, *z*+1; (ii) *x*, *y*−1, *z*; (iii) *x*, *y*, *z*+1; (iv) −*x*, *y*−1, −*z*+1.
